# Self-Rating Recovery in Forensic Settings: Associations Between Patients Views of Their Own Recovery, and Measures of Violence Risk and Symptoms

**DOI:** 10.1192/bjo.2022.251

**Published:** 2022-06-20

**Authors:** Caroline Synnott, Catherine Rock, Hania Amin, Eimear Ni Mhuricheartaigh, Harry G Kennedy, Mary Davoren

**Affiliations:** 1 National Forensic Mental Health Service, Central Mental Hospital, Dundrum, Dublin, Ireland; 2 Trinity College, Dublin, Ireland

## Abstract

**Aims:**

The aim of this study was to ascertain the correlations between patients’ views of their recovery and clinicians’ views of patients recovery, symptoms and risk, in a cohort of patients in the National Forensic Service Dundrum (NFMHS).

**Methods:**

A cross sectional study was performed of all inpatients in the NFMHS Dundrum. The self-rated Dundrum tool was offered to all 96 in-patients and completed by 64. Clinican rated measures of violence risk (HCR-20), programme completion (Dundrum-3), recovery (Dundrum-4), symptoms (PANSS) and functioning (GAF MIRECC) were rated. ANOVA and concordance ratings were calculated using SPSS

**Results:**

A total of 64 patients agreed to participate, of whom 10 were female. The self-rated Dundrum-3 correlated with the staff rated Dundrum-3 (0.471, p < 0.001). The self-rated Dundrum-4 correlated with the staff rated Dundrum-4 (0.373, p = 0.003). The self-rated Dundrum-3 correlated with the HCR-20 total (0.0352, p = 0.005), HCR-C (0.3677, p = 0.004), and HCR-R (0.301, p = 0.018). The self-rated Dundrum-3 correlated significantly with GAF occupational (-0.273, p = 0.48), symptomatic (-0.299, p = 0.03). The self-rated Dundrum-4 correlated only with the GAF symptomatic (-0.333, p = 0.05). The self-rated Dundrum-3 correlated with PANSS positive (0.457, p = 0.001), PANSS negative (0.514, p < 0.001), PANSS general (0.395, p = 0.004) and PANSS total (0.352, p = 0.005). The self-rated Dundrum-4 correlated with PANSS positive (0.356, p = 0.01) and PANSS negative (0.413, p = 0.002).

**Conclusion:**

There was good correlation between patient and clinician ratings of programme completion and recovery. Patient self-ratings of programme completion and recovery correlated with staff ratings of functioning and symptoms. The directions of agreement were correct

